# Antibacterial and Antifungal Activity of Metabolites from Basidiomycetes: A Review

**DOI:** 10.3390/antibiotics13111026

**Published:** 2024-10-31

**Authors:** Valeria Lysakova, Larissa Krasnopolskaya, Maria Yarina, Mayya Ziangirova

**Affiliations:** FSBI Gause Institute of New Antibiotics, Bol’shaya Pirogovskaya Str., 11, 119021 Moscow, Russia; lmkrasnopolska@gmail.com (L.K.); maria.s.yarina@gmail.com (M.Y.); ziangirova.m@gmail.com (M.Z.)

**Keywords:** basidiomycetes, metabolites, extracts, fruiting bodies, mycelium, culture liquid, antibiotics, antimicrobial activity, antifungal activity

## Abstract

**Background/Objectives**: The search for new antimicrobial molecules is important to expand the range of available drugs, as well as to overcome the drug resistance of pathogens. One of the promising sources of antibacterial and antifungal metabolites is basidial fungi, which have wide biosynthetic capabilities. **Methods**: The review summarized the results of studying the antimicrobial activity of extracts and metabolites from basidiomycetes published from 2018–2023. **Results**: In all studies, testing for antibacterial and antifungal activity was carried out in in vitro experiments. To obtain the extracts, mainly the fruiting bodies of basidiomycetes, as well as their mycelia and culture liquid were used. Antimicrobial activity was found in aqueous, methanol, and ethanol extracts. Antimicrobial metabolites of basidiomycetes were isolated mainly from the submerged culture of basidiomycetes. Metabolites active against Gram-positive and Gram-negative bacteria and mycelial and yeast-like fungi were identified. **Conclusions**: Basidiomycete extracts and metabolites have shown activity against collectible strains of bacteria and fungi and multi-resistant and clinical strains of pathogenic bacteria. The minimum inhibitory concentration (MIC) values of the most active metabolites ranged from 1 to 16.7 µg/mL.

## 1. Introduction

The search for new antimicrobial molecules is important for expanding the range of available drugs. This is especially relevant because of the constant increase in microorganisms with drug resistance. Therefore, the search for new molecules with antibacterial and/or antifungal properties is relevant. Fungi have high biosynthetic abilities. They are an important source of biologically active compounds with a diverse chemical structure. A significant chemical and functional diversity of metabolites is characteristic of basidial fungi, representatives of the Basidiomycota division, which includes more than 40,000 species [1]. This diversity is based on the use of shorter biosynthetic pathways by basidiomycetes compared to ascomycetes. Therefore, the processes of anabolism occur in basidiomycetes with lower energy costs. Metabolites of basidial fungi exhibit antibacterial, antifungal, antiviral, immunomodulatory, antitumor, anti-inflammatory, antioxidant, and other properties [2,3,4]. The chemical nature of biologically active compounds synthesized by basidiomycetes varies. Basidiomycetes can produce substances of various chemical natures, such as polyphenols, polysaccharides, proteins, lipids, terpenes, terpenoids, alkaloids, and others [5,6,7,8]. Many of these compounds synthesize only basidiomycetes, unlike fungi from other taxa or bacteria. Antimicrobial metabolites are classified as secondary metabolites, which in the vast majority of cases, are not necessary for the existence of their producers. In contrast to primary metabolites, secondary metabolites are individually produced compounds, often specific to one species or a limited group of species [9]. The diversity of secondary metabolites is based on the fact that they are often formed as families of compounds that are similar in chemical structure and share the same biosynthesis pathway.

Basidiomycetes from different ecological trophic groups grow in soil, on wood, and in other various multicomponent substrates, and parasitize on plants and animals [10]. During the long coevolution, basidiomycetes developed their own protective mechanisms, including specialized chemical protection. Secondary metabolites with antibiotic activity act as weapons against competing organisms occupying and consuming the same substrate, as well as signaling molecules for interspecific and intraspecific communication [11].

It is known that the first antibiotic, penicillin, was isolated in 1928 by A. Fleming from the culture of the fungus *Penicillium notatum*. The screening and study of antimicrobial metabolites of ascomycetes is continuing. Currently, ascomycetes are being intensively studied. As a result, several antimicrobial compounds have been isolated from them, for example, talaromycins A and B (*Talaromyces* sp.), cyclopeptides (*Phomopsis* sp. and *Alternaria* sp.), and curvulamine (*Curvularia* sp.) [12,13,14]. Since the 1950s, actinomycetes (phylum Actinomycetota) have been actively studied as producers, which led to the discovery of such promising antibiotics as streptomycin, chloramphenicol, tetracyclines, and polyenes [15].

Metabolites of basidiomycetes with antimicrobial activity have been extensively studied since the 1940s. The research was initiated by biological laboratories in New York and Oxford [16,17], and after some time, the geography of research expanded significantly. During the first ten years, more than 2000 species of basidiomycetes were studied [17,18]. Many of these species produced compounds that could inhibit the growth of bacteria and/or fungi. By 2013, more than 280 species of basidial fungi with antibacterial activity against both Gram-positive and Gram-negative bacteria were identified [19,20]. Several of these compounds had significant potential for use in medicine and sustainable agriculture [21]. One of the most promising compounds was pleuromutilin, synthesized from species of the genera *Clitopilus* and *Omphalina* [16,22]. This compound, which had a unique mechanism of action, exhibited high activity against Gram-positive bacteria [23]. The pleuromutilin derivatives tiamulin and valnemulin are used in veterinary medicine, and retapamulin is approved for the treatment of human skin diseases. The first systemic antibiotic of the pleuromutilin class, lefamulin, was approved for the treatment of community-acquired bacterial pneumonia [24]. The second group of compounds that have proven highly effective in their use are strobilurins, which had antifungal activity and were discovered in 1977 as metabolites of the basidiomycete *Strobilurus tenacellus* [25]. Later, substances of this class were found in other species, for example *Mucidula mucida*, *Xerula longipes*, *Xerula pudens* [26], and *Oudemansiella canarii* [27]. Strobilurins obtained by chemical synthesis are used in the practice of crop production. Sales of strobilurin preparations account for 23–25% of the global fungicide market [28].

The need for new antibiotics has increased due to the emergence and spread of pathogens resistant to existing antibiotics, especially multidrug-resistant pathogens. This led to an intensification of the search for new active molecules among the metabolites of various producers, including basidiomycetes [29,30]. According to the PubMed database, the request “Basidiomycota + antibacterial”, yielded 348 studies conducted between 2000 and 2013 years, and 612 works were conducted between 2014 and 2023. The query “Basidiomycota + antimicrobial” for the same time interval yielded 1597 and 2032 studies, respectively.

In this review, we summarized data from the literature on the antibacterial and antifungal activities of extracts and individual metabolites of basidial fungi published by researchers between 2018 and 2023. Typical cultures of microorganisms, clinical isolates, drug-resistant strains, and strains capable of biofilm formation were used as test objects in the cited articles.

## 2. Antimicrobial Activity of Basidial Fungi Extracts

The general scheme of natural antibiotics screening was the same for all producers. During the first stage, researchers studied extracts of the analyzed biomaterial. Organic solvents are used as extractants, for example, ethyl acetate, ethanol, acetone, etc., as well as hot and cold water [31,32,33,34]. The obtained materials are studied mainly in in vitro experiments. The most commonly used method was the disk diffusion method, which is based on the antibiotic’s ability to diffuse from the carrier into a solid nutrient medium. Serial dilution methods are also used to obtain the minimum inhibitory concentration (MIC), minimum bactericidal concentration (MBC), and minimum fungicidal concentration (MFC). Researchers also used other methods, for example, the method based on the release of pyocyanin. As test objects, Gram-positive and Gram-negative bacteria and mycelial and yeast-like fungi are used. These microorganisms could be saprotrophs, human and/or animal pathogens, or phytopathogens. The activity was studied against both commonly used species of test organisms, for example, *Staphylococcus aureus*, *Pseudomonas aeruginosa*, *Candida albicans*, etc., and rarer ones, for example, *Eggerthella lenta*, *Vibrio parahaemolyticus*.

The most active producers were selected from each research. The selection criteria were MIC values less than 2 mg/mL (2000 µg/mL) and values of growth inhibition zones greater than 15 mm. The numeric activity values are presented in Table 1 and Table 2.

**Figure 1:**
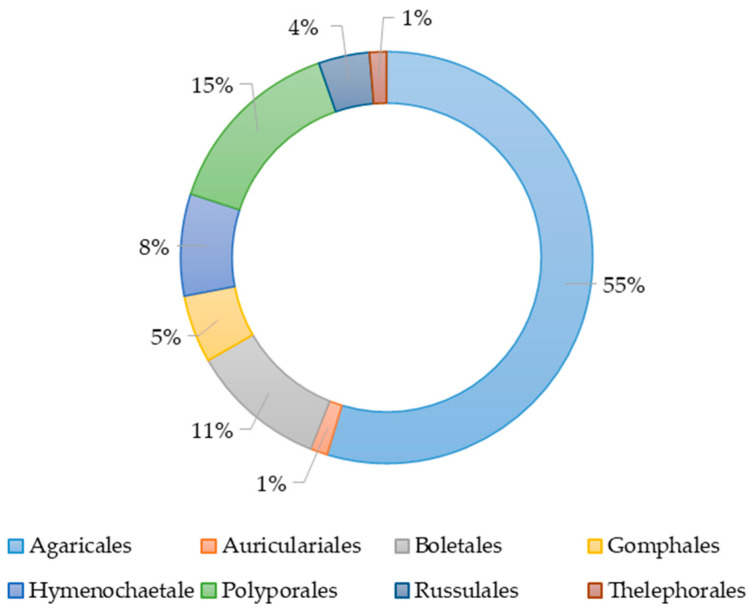
The percentage of basidiomycete orders’ representatives in studies on the antimicrobial activity of their various extracts.

The species of basidiomycetes used in the study of their extracts antimicrobial action belonged to eight orders, namely Agaricales, Polyporales, Boletales, Hymenochaetales, Russulales, Auriculales, Gomphales, and Thelephorales (Figure 1). More than half of all studies had been conducted with species from the order Agaricales. The orders Auriculares and Thelephorales were represented by a single species. The species belonging to the objects was determined on the basis of morphological characteristics or sequencing.

### 2.1. Basidiomycetes Orders and Activities of Extracts

The study of species belonging to the order Agaricales showed that activity against Gram-positive bacteria was found in extracts of fruit bodies of *Agaricus bisporus*, *Lentinula edodes* [35,36], *Cortinarius traganus*, *Cyclocybe aegerita* [37], *Cyclocybe cylindracea* [38], peptide extracts of culture liquid *Hypsizygus ulmarius* and *Pleurotus eryngii* [39], fruit bodies of *Gymnopilus junonius*, *Mycena* sp. and *Tricholoma equestre* [40], and *Termitomyces* spp. [41], and fruit bodies and mycelium of *Tricholosporum goniospermum* [42]. *Termitomyces* spp extract inhibited the growth of methicillin-resistant *S. aureus* ATCC 33591 (MRSA) [41].

Fruit body extracts of *A. bisporus*, L. edodes [35], *C. cylindracea* [38], *Cortinarius nanceiensis*, *Cortinarius reverendissimus* [43], *G. junonius*, *Mycena* sp., *T. equestrie* [40], and *Termitomyces* spp. [41], and extracts of fruit bodies and mycelium of *T. goniospermum* [42] were active against Gram-negative bacteria.

Antifungal activity was found in extracts of the fruit bodies of *Amanita proxima*, *Amanita virosa*, and *Trametes quercina* [44], mycelium and culture fluid of *Favolaschia calocera* [45], fruit bodies of *Termitomyces* spp. [41], and fruit bodies and mycelium of *T. goniospermum* [42]. In [44], the activity against only phytopathogenic fungi was studied, including *Aspergillus niger*; *Fusarium oxysporum* f.sp. *albedinis*; and *Verticillium dahlia*.

A study of *Auricularia* spp. (order Auriculares) has shown that fruit body extracts exhibited antibacterial and antifungal activity. Antibacterial activity had been found against Gram-negative and Gram-positive bacteria, including MRSA ATCC 33591 [41].

In the study of fungal species belonging to the order Boletales, the antimicrobial effect of fruit body extracts was evaluated. Activity against Gram-positive bacteria was detected in extracts of *Boletus edulis*, *Neoboletus luridiformis* [46], *Gyroporus castaneus*, and *Rubroboletus lupinus* [37]. The growth of Gram-negative bacteria was inhibited by extracts of *B. edulis* and *N. luridiformis* [46]. *Xerocomus* sp. fruit body extract had antifungal activity [44]. Activity against drug-resistant strains of pathogenic bacteria has been found in species from the order Boletales. Aqueous and methanol extracts of *B. edulis* and *N. luridiformis* inhibited the growth of clinical multidrug-resistant strains of *S. aureus* (MRSA) MJMC 027, *P. aeruginosa MJMC 540*, *Escherichia coli* CETC 434 [46]. Extracts of *G. castaneus* and *R. lupinus* were active against MRSA B5284 [37]. The aqueous extract of *B. edulis* effectively suppressed the formation of *S. aureus* CECT 976 (78%) and *E. coli* CECT 434 (94%) biofilms. According to the authors, the antibacterial activity of *B. edulis* is associated with phenolic nature metabolites, for example a protocatechuic acid, found in an aqueous extract, and 2,4-dihydroxybenzoic acid, found in a methanol extract [46].

In the work of Clericuzio et al., samples of the orders Gomphales (*Ramaria pallida, Ramaria parabotrytis*, *Ramaria pallidosaponaria*, and *Ramaria flavescens*) and Thelephorales (*Hydnellum spongiosipes*) were studied. All fruit body extracts of the listed basidiomycetes species had the ability to inhibit the growth of *P. aeruginosa* and/or the release of pyocyanin. The most active extracts were obtained from *R. parabotrytis* and *H. spongiosipes* [43].

Among the specimens of the order Hymenochaetales, species were identified whose extracts of biomass and/or culture liquid had antimicrobial activity. Extracts of fruit bodies of *Fuscoporia torulosa* [47], *Fuscoporia ferruginosa*, and *Phellinus tuberculosus* [48] and of culture liquid and mycelium of *Inonotus pachyphloeus* [45] were active against Gram-positive and Gram-negative bacteria. Extracts of *F. torulosa* fruit bodies [47] and *I. pachyphloeus* culture liquid [45] had antifungal activity. Extract of *F. torulosa* was active against the phytopathogenic fungi *Sclerotinia sclerotiorum* and *Verticillium* sp. resistant to amphotericin B and itraconazole [47].

Members of the order Polyporales exhibited various antimicrobial activities. Extracts of the fruit bodies of *Bjerkandera adusta*, *Lentinus squarrosulus* [38], and *Fomitopsis officinalis* (*Laricifomes officinalis*) [49] as well as of mycelium and culture fluid of *Hexagonia* sp. and *Skeletocutis nivea* [45] were active against Gram-positive bacteria. Of these, extracts of *F. officinalis* [49], *Hexagenia* sp. (culture fluid) [45], and *L. squarrosulus* [38] were active against Gram-negative bacteria. Antifungal activity was found in *F. officinalis* [49], *Lentinus crinitus* [50], and *S. nivea* (mycelium) extracts [45]. The ethanol extract of *B. adusta* showed a high content of chlorogenic acid, with which researchers associated the high antimicrobial activity of this extract [38].

### 2.2. Sources of Antimicrobial Metabolites

In most studies (75%) the antimicrobial effect of fruit body extracts was investigated. The antimicrobial activities of basidiomycetes fruit bodies and mycelium were compared in two studies. The objects of the study were extracts of fruit bodies and mycelium of *L. crinitus* obtained under conditions of submerged cultivation and solid-state fermentation [50] and extracts of fruit bodies and mycelium of *T. goniospermum* grown on agar dense medium [42]. The results of both studies showed that mycelium extracts had the greatest activity. The only exception was the effect of methanol extracts of the *T. gonospermum* fruit body and mycelium against *Bacillus cereus*.

In Flores et. al.’s article, differences were revealed in the antimicrobial activity of the extracts obtained from three different parts of the *F. officinalis* fruit body, including the apical and median parts, as well as the hymenium. The extract of the apical part showed the greatest activity against the Gram-negative bacterium *P. aeruginosa* and the dermatophyte fungi *Arthroderma currei* and *Trichophyton tonsurans*. This extract contained the largest amount of phenolic nature metabolites [49].

### 2.3. The Extractants Impact

**Figure 2:**
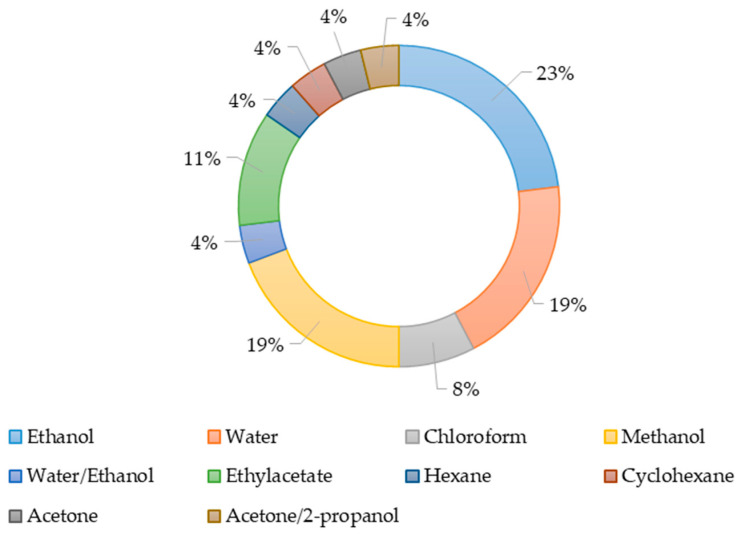
The use of various solvents to produce extracts.

The solvents used to produce the extracts are shown in Figure 2. Ethanol, methanol, and water were most often used as extractants during the study of the antimicrobial activity of extracts. Obviously, the effectiveness of various solvents is related to the chemical nature of the active substance. The presence of active substances in producer biomass depends on their taxonomic affiliation, geographical and seasonal features of growth for wild specimens, or the growing conditions in the laboratory.

The results of comparing the antimicrobial activity of extracts of *Auricularia* spp. and *Termitomyces* spp. fruit bodies obtained using hot water, ethanol, and chloroform were the same for both species. The greatest antibacterial and antifungal (*C. albicans*) activity was found in aqueous extracts against Gram-positive (MRSA, *S. aureus*) and Gram-negative (*E. coli*, *Klebsiella pneumoniae*) bacteria [41]. Similar results were obtained during the testing of methanol and aqueous extracts of *B. edulis* and *T. luridiformis* fruit bodies. Antibacterial activity was higher in aqueous extracts [46]. However, a comparison of cyclohexane, chloroform, ethanol, and aqueous extracts of *N. luridiformis* fruit bodies in the study [37] showed that cyclohexane extract had greater antimicrobial activity. Ethanol extracts of *A. bisporus* fruit bodies had antimicrobial activity [35,36], and ethyl acetate extracts had no activity [44]. It should be noted that the differences could be related to the study of fruit bodies of different strains.

The fruit bodies of *F. ferruginosa* and *P. tuberculosus* were extracted with several solvents, including water (cold and hot), methanol, and ethanol. Both extracts prepared using hot water did not have antibiotic activity, as well as the *F. ferruginosa* extract obtained with cold water. Ethanol and methanol extracts of fruit bodies of both types of basidiomycetes had similar activity [48].

Basidiomycetes metabolites with antimicrobial properties can be found in fruiting bodies, mycelia, and/or culture liquids. The fruiting bodies used in most studies are collected in nature. Basidiomes grown in culture were rarely used. It is not always possible to reproduce the results of studies of fruiting bodies collected in nature, since the climate and geographic location of the growth place has a serious impact on the metabolome. Reproducibility and reliability of obtained results are ensured by the use of pure cultures of basidiomycetes and their submerged cultivation. Basidiomycetes demonstrated antibacterial and antifungal activity at the level of crude extracts, which makes it possible to identify the most promising cultures for the isolation of active metabolites. The aqueous extract of the fruiting bodies of *B. edulis* showed a MIC of 7.81 µg/mL for *P. aeruginosa* [46]. In the aqueous extract of the apical part of *F. officinalis*, the MIC was 3.86 µg/mL for *E. coli* [49]. Extracts of both the mycelium and culture liquid of *F. calocera* showed antifungal action against *Candida tenuis*, with MIC values of 2.34 and 4.69 µg/mL, respectively. Activity against *Mucor plumbeus* was lower (MIC > 37.50 µg/mL). Antibacterial activity against *Bacillus subtilis* was demonstrated by extracts of mycelium and culture liquid *S. nivea* with MICs of 9.38 and 4.69 µg/mL, respectively [45]. The antimicrobial effect has been established in extracts of various natures, such asaqueous, methanol, and ethanol extracts. In addition, the extracts demonstrate activity against clinical and resistant strains of microorganisms. Later, these data could be used by researchers to improve the processes of both screening and isolation of biologically active metabolites.

**Table 1:** Antimicrobial activity of basidiomycetes extracts (MIC).

Species	Fungi Material	Extractant	Minimum Inhibitory Concentration (MIC), µg/mL						SEQ *	Reference
			Antibacterial Activity				Antifungal Activity			
			Gram-Positive Bacteria		Gram-Negative Bacteria					
*Auricularia* spp.	Fruiting bodies	Chloroform	MRSA	1000	*K. pneumoniae*	1330	*C. albicans*	1330	No	[41]
			*S. aureus*	1000	*P. aeruginosa*	1670	*C. parapsilosis*	1330		
		70% Ethanol	MRSA	1000	*E. coli*	1330	*C. albicans*	1330		
			*S. aureus*	830	*K. pneumoniae*	1000	*C. parapsilosis*	1000		
		Hot water	MRSA	1000	*E. coli*	1000	*C. albicans*	1000		
					*K. pneumoniae*	830				
			*S. aureus*	830	*P. aeruginosa*	1330	*C. parapsilosis*	830		
*Boletus edulis*	Fruiting bodies	Water	MRSA	31.25	*E. coli*	62.50	Not investigated		No	[46]
			*S. aureus*	15.63	*P. aeruginosa*	7.81				
		Methanol	MRSA	250	*P. aeruginosa*	125	Not investigated			
			*S. aureus*	125						
*Favolaschia calocera*	Culture liquid	Ethyl Acetate	Not detected		Not detected		*C. tenuis*	4.69	Yes	[45]
							*M. plumbeus*	75.00		
	Mycelium	Acetone	Not detected		Not detected		*C. tenuis*	<2.34		
							*M. plumbeus*	37.50		
*Fomitopsis officinalis*	Apical part of fruiting body	EtOH/Water 7:3	*B. cereus*	19.71	*E. coli*	3.86	*A. currei*	31.49	Yes	[49]
			*S. aureus*	31.49	*P. aeruginosa*	7.71	*T. tonsurans*	19.57		
	Median part of fruiting body		*B. cereus*	19.71	*E. coli*	7.71	*A. currei*	39.68		
			*S. aureus*	39.68	*P. aeruginosa*	125.99	*T. tonsurans*	31.49		
*Fuscoporia torulosa*	Fruiting bodies	Methanol	*B. cereus*	570–1130	*E. coli*	570–1130	*S. sclerotium*	570	Yes	[47]
							*Verticillium* sp.	570		
*Gyroporus castaneus*	Fruiting bodies	Cyclohexane	MRSA	125	Not detected		Not investigated		No	[37]
			*S. aureus*	125						
*Lentinula edodes*	Fruiting bodies	Ethanol	*S. aureus*	1560	Not detected		Not investigated		No	[36]
*Neoboletus luridiformis*	Fruiting bodies	Water	MRSA	62.5	*E. coli*	125	Not investigated		No	[46]
			*S. aureus*	250	*P. aeruginosa*	31.25				
		Methanol	MRSA	250	*P. aeruginosa*	250	Not investigated			
			S. aureus	250						
*Phellinus tuberculosus*	Fruiting bodies	Ethanol	*S. aureus*	700	Not detected		Not detected		No	[48]
			*S. mutans*	1560						
*Rubroboletus lupinus*	Fruiting bodies	Cyclohexane	MRSA	125	Not detected		Not investigated		No	[46]
			*S. aureus*	250						
*Skeletocutis nivea*	Culture liquid	Ethyl Acetate	*B. subtilis*	9.38	Not detected		Not detected		Yes	[45]
	Mycelium	Acetone	*B. subtilis*	4.69			*M. plumbeus*	300		
*Termitomyces* spp.	Fruiting bodies	70% Ethanol	MRSA	830	*E. coli*	1000	*C. albicans*	1000	No	[41]
			*S. aureus*	670	*K. pneumoniae*	1000	*C. parapsilosis*	1000		
		Hot water	MRSA	830	*E. coli*	830	*C. albicans*	830		
			*S. aureus*	670	*K. pneumoniae*	830	*C. parapsilosis*	830		
*Tricholosporum goniospermum*	Mycelium	Ethyl Acetate	*B. subtilis*	78	*E. coli*	99	*C. albicans*	51	Yes	[42]
			*B. cereus*	99			*T. tonsurans*	39		
	Fruiting bodies	Methanol	*B. cereus*	99	*E. coli*	198	*C. albicans*	198		

* SEQ—sequencing to determine the taxonomic position of a culture. Not investigated—no testing had been conducted against this group of microorganisms. Not detected—no activity based on the results of article.

**Table 2:** Antimicrobial activity of basidiomycetes extracts (Inhibition zone).

Species	Fungi Material	Extractant	Inhibition Zone, mm						SEQ *	Reference
			Antibacterial Activity				Antifungal Activity			
			Gram-Positive Bacteria		Gram-Negative Bacteria					
*Amanita proxima*	Fruiting bodies	Ethyl Acetate	Not investigated		Not investigated		*A. niger*	33.33	No	[43]
							*F. oxysporum*	26.33		
							*V. dahliae*	16.67		
*Amanita virosa*	Fruiting bodies	Ethyl Acetate	Not investigated		Not investigated		*A. niger*	31.50		
							*F. oxysporum*	33.50		
							*V. dahliae*	21.67		
*Bjerkandera adusta*	Fruiting bodies	Ethanol	*S. pneumoniae*	17	Not detected		Not detected		Yes	[38]
			*S. aureus*	15						
*Cyclocybe cylindracea*	Fruiting bodies	Ethanol	*S*. *pneumoniae*	20	*E*. *coli*	17	Not detected		Yes	[38]
					*P*. *aeruginosa*	17				
*Gymnopilus junonius*	Fruiting bodies	Methanol	*E. faecalis*	17	*V. parahaemolyticus*	25	Not investigated		No	[45]
			*E. lenta*	26						
*Mycena* sp.	Fruiting bodies	Methanol	*E. lenta*	15	Not investigated		Not investigated			
*Trametes quercina*	Fruiting bodies	Ethyl Acetate	Not investigated		Not investigated		*A. niger*	14.50	No	[43]
							*F. oxysporum*	36.83		
							*V. dahliae*	24.00		
*Tricholoma equestre*	Fruiting bodies	Methanol	*E. faecalis*	17	*V. parahaemolyticus*	21	Not investigated		No	[45]
			*E. lenta*	17						

* SEQ—sequencing to determine the taxonomic position of a culture. Not investigated—no testing had been conducted against this group of microorganisms. Not detected—no activity based on the results of article.

## 3. Antibacterial and Antifungal Metabolites of Basidial Fungi

During the search for a metabolite with antibacterial or antifungal properties, it is necessary to study the original extract and isolate the individual substance that is responsible for the activity. To carry this out, various physicochemical methods are used, so high-performance liquid chromatography (HPLC) and flash chromatography are often used to separate extracts. At the same time, nuclear magnetic resonance (NMR) methods (1H, 13C, heteronuclear multiple quantum coherence (HMQC), and others), mass spectrometry (for example electrospray ionization mass spectrometry (ESIMS)), and ultraviolet (UV) and infrared (IR) spectroscopy are mainly used to determine the structure of target metabolites.

The use of these methods and the ever-expanding databases, which store spectral and other physical data, significantly improved the possibilities and efficiency of purification and the identification of target compounds.

However, phenomena, such as the decomposition of compounds, their loss during separation, or the possibility that mixtures could have effects not detectable in purified/individual components, could ultimately prevent the identification of secondary metabolites with antibiotic properties [51]. Reproducibility issues could arise when researchers continue or repeat studies to identify metabolites isolated from fruiting bodies collected in the environment.

This review presented the results of the past few years’ research on the pursuit of new antimicrobial compounds from basidiomycetes, as well as on the verification of the target activity of already-known natural metabolites.

The most active substances were selected from the articles devoted to the isolation and identification of active antibacterial and antifungal metabolites. The numerical values of MICs and growth-suppression zones are presented in Table 3 and Table 4.

**Figure 3:**
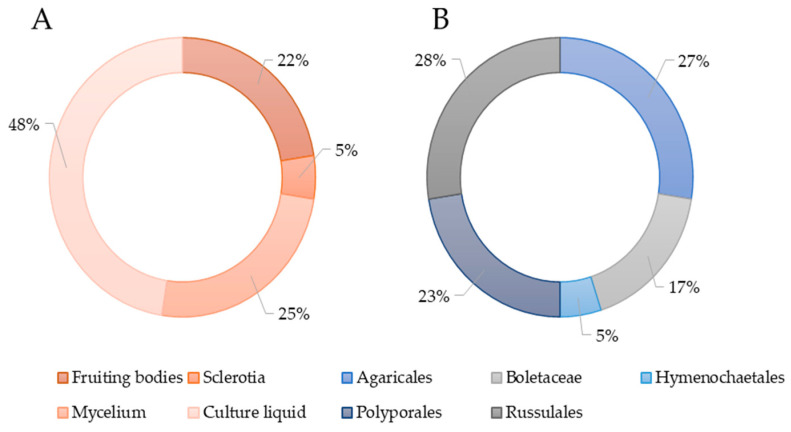
(**A**) Sources of antimicrobial molecules of basidiomycetes. (**B**) The percentage of basidiomycete orders representatives in studies on the isolation of antimicrobial molecules.

The isolation of individual biologically active metabolites in most studies was carried out from the submerged culture of the producer (Figure 3A). From a taxonomic point of view, the identified producing strains belonged to species included in the orders Agaricales, Boletales, Hymenochaetales, Russulales, and Polyporales (Figure 3B).

### 3.1. Order Agaricales

**Figure 4:**
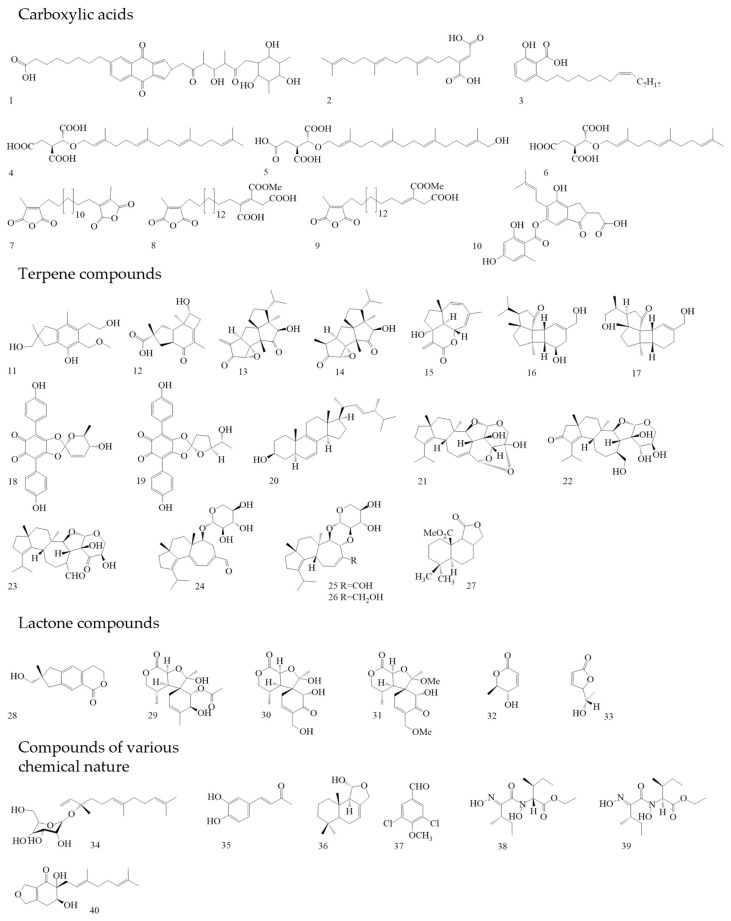
Basidiomycetes metabolites with antimicrobial activity.

The organic acid naphthoquinone derivative Griseococcin (Figure 4(1)) was isolated from the culture liquid of *Bovistella radicata*. This substance had antibacterial and antifungal activity [52,53]. Woo et al. isolated three substances from the culture liquid of the basidiomycete *Schizophyllum commune*, the new nerolidol mannoside mannonerolidol, and the already-known organic acids: schizostatin [54] and nerolidol [55]. Using a disc diffusion method with an amount of 50 µg per disc, antifungal activity against phytopathogenic fungi (*Rhizoctonia solani*, *Diaporthe* sp., *Botrytis cinerea*, and *Alternaria solani*), as well as the antibacterial activity against *B. subtilis* and *S. aureus*, was established. Schizostatin (Figure 4(2)) and mannonerolidol (Figure 4(34)) demonstrated antimicrobial activity [56].

Woo et al. isolated three compounds from *Coprinus rhizophorus* culture liquid using various types of liquid chromatography [57]. Two of these compounds (Figure 4(11),(12)) were new sesquiterpene molecules, and the third compound was identified as echinolactone D (Figure 4(28)), previously isolated from *Echinodontium japonicum* [58]. In [57], researchers investigated the antibacterial activity by the disc diffusion method. Sesquiterpene 1 (Figure 4(11)) showed no antibacterial activity at a dose of 200 µg/disc. Sesquiterpene 2 (Figure 4(12)), when applied at 40 µg per disc, showed antibacterial activity against *Staphylococcus epidermidis* and *Propionibacterium acnes*. At a dose of 200 µg per disc, echinolactone D showed activity against *Saccharomyces cerevisiae*. In addition, sesquiterpene 1, unlike the other compounds, showed antioxidant activity (ABTS radical-scavenging assay) [57]. Two terpenoids were isolated from the culture liquid of *Crinipellis rhizomaticola*. The compound crinipellin A (Figure 4(13)) showed antifungal activity against the phytopagenic fungi *Colletotrichum coccodes*, *Magnaporthe oryzae*, *B. cinerea*, and *Phytophthora infestans*. At the same time, crinipellin I (Figure 4(14)) had weak activity (MIC ≥ 250 µg/mL) compared with crinipellin A [59].

Sandargo et al. isolated tetraterpenes fulvoferruginins A–F from the extract of the culture liquid *Marasmius* spp. Antifungal, antibacterial, and cytotoxic activities have been tested for these substances. Only fulvoferruginin A (Figure 4(15)) demonstrated antifungal activity; antibacterial activity was absent in all substances. Also, only this compound showed strong cytotoxic activity against various cancer cell lines (The half maximal inhibitory concentration (IC_50_) values range from 0.06 to 0.6 µg/mL). However, a similar activity was present against mouse fibroblasts (IC_50_: 0.7 µg/mL). The authors of the work suggested that the lack of activity in other compounds could be due to the absence of the α-methylene lactone unit in all compounds except fulvoferruginin A [60]. It should be mentioned that this compound was previously obtained from other fungi, including *Marasmius fulvoferrugineus* and *Gymnopus* sp. [61,62]. Two new diterpenoids were isolated from the culture liquid of *Psathyrella candolleana*, namely psathyrins A (Figure 4(16)) and B (Figure 4(17)). Both compounds demonstrated activity against *S. aureus* and *Salmonella enterica* [63,64].

### 3.2. Order Boletales

From the fruiting bodies of the basidiomycete *Caloboletus radicans*, three new lactone compounds similar in structure to cyclocalopines were isolated, as well as seven already-known molecules. The antibacterial activity of these compounds against several MRSA strains has been tested. 8-deacetylcyclocalopin B (Figure 4(29)) demonstrated higher activity than the positive control (norfloxacin) in relation to MRSA strains ATCC 25,923 and SA-1199B with an MIC value of 16 µg/mL. Cyclocalopin-A-15-ol (Figure 4(30)) also showed activity against these strains. 12,15—dimethoxycyclocalopin A (Figure 4(31)) had less activity than the previous compounds. It has been suggested that the activity is connected with the absence of a free hydroxyl group at the C_12_ position. These compounds had no cytotoxic effect on the prostate cancer cell line PC3 and hepatoblastoma cell line HepG2 [65]. For the first time, calopins were isolated from the fruiting bodies of *Boletus calopus*, but no study of biological activity was carried out [66]. 

It was previously shown that *Tapinella atrotomentosa* fruiting body extract demonstrated antimicrobial activity [67]. Later, two compounds with a lactone structure (osmundalactone and 5-hydroxy-hex-2-en-4-olide) and two compounds with a terpenylquinone skeleton (spiromentin C and spiromentin B) were isolated from the fruiting bodies extract of this basidiomycete. These substances were tested against Gram-negative bacteria with different types of resistance mechanisms. Osmundalactone (Figure 4(32)) demonstrated activity against a multi-resistant strain of *A. baumannii* SZMC 24075 and a strain of *E. coli* SZMC 24090 producing beta-lactamases. 5-hydroxy-hex-2-en-4-olide (Figure 4(33)) had activity against *A. baumannii*, *E. coli,* and *Moraxella catarrhalis* ATCC 25238. Spiromentin C (Figure 4(18)) showed an antibacterial effect on the same microorganisms. Spiromentin B (Figure 4(19)) demonstrated activity only against *E. coli* [68].

### 3.3. Order Hymenochaetales

Osmundacetone and four other compounds were isolated from sclerotia *Inonotus nidus-pici*; only two molecules had antibacterial activity. Osmundacetone (Figure 4(35)) showed low activity against the Gram-negative bacterium *Aliivibrio fischeri,* with an MIC value of 93.8 µg/mL. Ergost-6,8,22-trien-3β-ol (Figure 4(20)) demonstrated action against the Gram-positive bacteria *B. subtilis* and *Rhodococcus fascians*. Both compounds demonstrated cytotoxic activity. The Osmudacetone IC_50_ was 57.5 µM for MESSA (uterine sarcoma cell line) and 101.3 µM for A431 (human epi-dermoid carcinoma cell line). Ergost-6,8,22-tsrien-3β-ol demonstrated an IC_50_ equal to 41.9 µM (MESSA) and 83.2 µM (A431) [69]. Osmundacetone was previously isolated from the basidiomycete *Fuscoporia torulosa* [70].

### 3.4. Order Polyporales

Four anacardic acid derivatives were isolated from the methanol extract of *Aurantiopileus matanensis* fruiting bodies. Merulinic acid C (Figure 4(3)) showed activity against multidrug-resistant strains of *E. faecium* 72432, 72772, 72827, 72723, and 72948. The MIC was 16 µg/mL for all these strains. In further experiments, it was proven that merulinic acid C worked on the cytoplasmic membranes of bacteria, destroying them. In addition, it has been proven that gentamicin and merulinic acid C can act synergistically [71]. Chepkirui et.al. isolated seven new compounds from the mycelium *Microporus* sp. These compounds can be attributed to both diterpenes and organic acids. The results of the verification of antibacterial and antifungal activity showed that microporenic acids A (Figure 4(4)), D (Figure 4(5)), and E (Figure 4(6)) have the target properties. Microporenic acid E showed the greatest antibacterial activity against *M. luteus*. Microporenic acid D was the most active against *S. aureus*. Microporenic acids A and D showed a dose-dependent ability to inhibit the formation of *S. aureus* DSM 346 and *C. albicans* DSM 1665 biofilms. Microporenic acid A demonstrated weak cytotoxic activity against the mouse fibroblast cell lines L929 and HeLa (KB-3.1). Other compounds did not show cytotoxic activity [72].

From mycelium and culture liquid extracts of *Skeletocutis* sp. twelve previously unknown acid skeletocutins A-L (1–5 and 7–13) were isolated, as well as the already-known tyromycin A (6) [73]. MICs were determined for all complex carboxylic acids towards various microorganisms. All compounds had no activity against Gram-negative bacteria and fungi. However, nine substances had activity against Gram-positive bacteria, including MRSA DSM 11822. The most active compounds were skeletocutin A (Figure 4(7)); skeletocutin I (Figure 4(8)); and tyromycin L (Figure 4(9)). In addition to the antibacterial activity, antiviral activity against the Hepatitis C virus (HCV) was also tested. Tyromycin A inhibited the virus, with an IC_50_ value of 6.6 µM. Skeletocutins I and L also demonstrated weak cytotoxic activity (IC_50_: 84 and 73.3 µg/mL, respectively) against HeLa (KB 3.1) [74].

Pathompong et al. studied two basidiomycetes, namely *Cerrena* sp. nov and *Perenniporia centrali-africana*. From various extracts of the mycelium and cultural liquid of the first mushroom, three new drimane-type sesquiterpenoids were obtained, and from the second, two drimane-type sesquiterpenes were obtained, as well as the well-known compound isodrimenediol (Figure 4(36)) [75]. Only isodrimenediol had activity against *Mucor hiemalis* and *Rhodoturula glutinis*. This compound also showed cytotoxic activity on the cell lines L929 (mouse fibroblasts) (IC_50_—33 µg/mL) and A549 (adenocarcinomic human alveolar basal epithelial cells) (IC_50_—16 µg/mL) [76]. The volatile compound 3,5-dichloro-4-methoxybenzaldehyde (Figure 4(37)) was isolated from *Porostereum spadiceum* mycelium. At a concentration of 100 µg/mL, this substance inhibited the growth of phytopathogenic bacteria and fungi: *Clavibacter michiganensis subsp*., *R. solanacearum*, *Alternaria brassicicola*, and *Colletotrichum orbiculare* [77].

### 3.5. Order Russulales

Confluenines A–F (new N-oxidized l-isoleucine derivatives) have been isolated from *Albatrellus confluens*. The compounds were tested for antimicrobial activity against various bacteria. Confluenines E (Figure 4(38)) and F (Figure 4(39)) demonstrated activity against *S. aureus*. At the same time, these substances did not have cytostatic activity against the HL-60 (promyelocytic leukemia), MCF-7 (human mammary duct adenocarcinomas), A-549 (lung carcinoma), SMMC-7721 (human hepatocarcinoma), and SW480 (colon adenocarcinoma) cells lines (IC_50_ > 40 µM) [78]. A study by Aqueveque et. al. showed that an acidic compound, sterenin D (Figure 4(10)), was isolated from the culture liquid of *Stereum hirsutum*, which had an antifungal effect on *B. cinerea* [79].

Sum et. al., in their study, isolated eight new terpene compounds (dentifragilins A–H)from *Dentipellis fragilis* mycelium [80], as well as two already-known ones, namely striatal D [81] and laxitextine A [82]. In the work of Sum and co-authors, dentifragilin A (Figure 4(21)) showed high activity against the Gram-positive bacteria *B. subtilis* and *S. aureus,* as well as antifungal activity against *M. hiemalis* and *Rhodotorula glutinis*. Dentifragilin D (Figure 4(22)) showed antibacterial action against *B. subtilis* and *S. aureus*. Striatal D (Figure 4(23)) had an antibiotic effect against many microorganisms, and high activity was noted against *B. subtilis*, *S. aureus*, *M. hiemalis*, *R. glutinis*, and *Schizosaccharomyces pombe* (MIC 4.2 µg/mL). The antifungal activity of this substance was on par with the control (nystatrin). Dentifragilins A-H, striatal D, and laxitextine A showed cytotoxic activity against the cell lines of mouse fibroblasts L929 and human endocervical adenocarcinoma KB3.1. The IC_50_ values of dentifragilin A were 5.8 µM and 2.2 µM, respectively. Striatal D has also shown activity against such cell lines as ovarian carcinoma SKOV-3, squamous cell carcinoma A549, and human breast adenocarcinoma MCF-7. The IC_50_ value for all three cell lines was 0.1 µM [80].

The other researchers isolated four compounds from the culture liquid *D. fragilis* using HPLC. One of them, dentipellin, was obtained for the first time. The others were glycosylated diterpenes erinacines A-C. These compounds were first isolated from the culture liquid of the basidiomycete *Hericium erinaceus* [83,84,85,86]. An earlier study of the neuroactivity of erinacines showed that these molecules trigger the production of the proteins BDNF and NGF, which are responsible for the growth of new neurons and the formation of neural connections [85,86]. In their work, Ha and co-authors found antimicrobial properties in all four obtained compounds. Dentipellin (Figure 4(40)) had activity against *Bacillus atrophaeus*, *B. cereus,* and *B. subtilis.* Erinacine A (Figure 4(24)) showed activity against the same test microorganisms and *S. epidermidis.* Erinacines B and C (Figure 4(25),(26)) suppressed the growth of *B. atrophaeus*, *S. epidermidis,* and *B. subtilis.* All compounds had antifungal properties and showed them against *B. cinerea*, *Colletotrichurn demantium, Diaporthe* sp., *F. oxysporum*, and *R. solani*. Among these substances, erinacine B had the highest activity and a wide spectrum. However, the activity of erinacine B against *P. acnes* and *S. epidermidis* was significantly lower than the erinacine C activity. The activity of erinacine C towards *B. subtilis* and *S. epidermidis* was comparable with the control (streptomycin) [83].

Also, four new drimane sesquiterpenoids were isolated from the submerged culture of *D. fragilis*. 10-Methoxycarbonyl-10-norisodrimenin (Figure 4(27)) had antimicrobial activity against *S. aureus* and *M. hiemalis*. This compound also showed cytotoxic activity against the mammalian cell line KB3.1 with an IC_50_ of 21.2 µM [87].

Basidiomycetes could synthesize highly active antimicrobial metabolites of various chemical natures, both at the vegetative and generative stages of their development. Most of these substances were sesquiterpenes, diterpenes, and terpenoids. Among the identified metabolites, both the new compounds and the previously known ones had targeted properties. Quantitative activity indicators of antimicrobial metabolites differed significantly. In nine published papers, the researchers presented the results of studying the cytotoxic properties of the identified metabolites, which increases their scientific value.

The most active compounds included merulinic acid C, 8-deacetyl-cyclocalopin B, erinacin B, dentifragiline A, fulvoferruginin A, microporenic acid E, tyromycin A, and osmudanlactone. 8-diacetyl-cyclocalopin B, cyclocalopin-A-15-ol, and skeletocutin A were active against various strains of MRSA. Osmundacetone and 5-hydroxy-hex-2-en-4-olide were active against Gram-negative bacteria with different resistance mechanisms. Merulinic acid C showed activity against multidrug-resistant strains of *E. faecium.* Microporenic acids A and D showed a dose-dependent ability to inhibit the formation of *S. aureus* and *C. albicans* biofilms. The activity of a number of the studied compounds was comparable to or exceeded the activity of positive controls. So, 8-deacetylcyclocalopin B demonstrated higher activity than the positive control (norfloxacin) in relation to MRSA (MIC: 16 µg/mL). Striatal D had an antifungal activity on par with the control nystatrin (MIC: 4.2 µg/mL), and the activity of erinacine C towards *S. epidermidis* was comparable with the control streptomycin (MIC: 10 µg/mL). If we compare the literature data on the activity of pleuromutilins (for example, tiamulin [88] and retapamulin [89]), then none of the compounds analyzed in this review could achieve the activity of pleuromutilins against *S. aureus* and MRSA.

**Table 3:** Antimicrobial activity of compounds isolated from basidiomycetes (MIC).

Species	Fungi Material	Compound	Minimum Inhibitory Concentration (MIC), µg/mL						SEQ *	Reference
			Antibacterial Activity				Antifungal Activity			
			Gram-Positive Bacteria		Gram-Negative Bacteria					
*Albatrellus confluens.*	Fruiting bodies	Confluenine E	*S. aureus*	29.3 **	Not detected		Not investigated		No	[78]
		Confluenine F	*S. aureus*	56.7 **	Not detected		Not investigated			
*Aurantiopileus mayanensis*	Fruiting bodies	Merulinic acid C	*B. subtilis*	16	Not detected		Not investigated		No	[71]
			*E. faceium*	16						
*Bovistella radicata*	Culture liquid	Griseococcin	*S. aureus*	62.5	*P. aeruginosa*	62.5	*T. mentagrophytes*	31.2	No	[52]
							*T. rubrum*	31.2		
*Caloboletus radicans*	Fruiting bodies	8-deacetylcyclocalopin B	MRSA	16	Not investigated		Not detected		No	[65]
		Cyclocalopin-A-15-ol	MRSA	64	Not investigated		Not detected			
		12,15–dimethoxycyclocalopin A	MRSA	128	Not investigated		Not detected			
*Crinipillis rhizomaticola*	Culture liquid	Crinipellin A	Not investigated		Not investigated		*B. cinerea*	31	Yes	[59]
							*C. coccodes*	1		
							*M. oryzae*	8		
							*P. infestans*	31		
*Dentipellis fragilis*	Mycelium	Dentifragilin A	*B. subtilis*	1	Not detected		*M. hiemalis*	16.7	Yes	[80]
			*S. aureus*	4.2			*R. glutinis*	16.7		
*Dentipellis fragilis*	Mycelium	Dentifragilin D	*B. subtilis*	16.7	Not detected		Not detected		Yes	[80]
			*S. aureus*	33.3						
		Striatal D	*B. subtilis*	1	Not detected		*M. hiemalis*	2.1		
							*R. glutinis*	1		
			*S. aureus*	2.1			*S. pombe*	4.2		
*Dentipellis fragilis*	Culture liquid	Erinacine A	*B. atrophaeus*	40	Not investigated		*C. demantium*	20	Yes	[83]
			*B. subtilis*	20						
		Erinacine B	*B. atrophaeus*	2.5	Not investigated		*B. cinerea*	10		
							*C. demantium*	20		
			*B. subtilis*	5			*Diaporte sp*.	5		
							*R. solani*	20		
		Erinacine C	*B. atrophaeus*	5	Not investigated		*B. cinerea*	20		
			*S. epidermidis*	10			*C. demantium*	20		
		Dentipellin	*B. atrophaeus*	80	Not investigated		*F. oxysporum*	20		
			*B. subtilis*	80						
*Dentipellis fragilis*	Culture liquid	10-Methoxycarbonyl-10-norisodrimenin	*S. aureus*	66.7	Not detected		*M. hiemalis*	66.7	Yes	[87]
*Inonotus nidus-pici*	Sclerotia	Osmundacetone	Not detected		*A. fischeri*	93.8	Not investigated		No	[69]
		Ergost-6,8,22-trien-3β-ol	*B. subtilis*	42	Not detected		Not investigated			
			*R. fascians*	168						
*Marasmius* spp.	Culture liquid	Fulvoferruginin A	Not detected		Not detected		*C. albicans*	8.3	Yes	[60]
							*M. hiemalis*	16.7		
							*R. glutinis*	33.3		
							*S. pombe*	66.7		
*Microporus* sp.	Mycelium	Microporenic acid A	*M. luteus*	37.5	Not detected		*M. plumbeus*	75	Yes	[72]
		Microporenic acid D	*B. subtilis*	37.5	Not detected		*C. tenuis*	37.5		
			*M. luteus*	18.8			*M. plumbeus*	75		
			*S. aureus*	75						
		Microporenic acid E	*B. subtilis*	18.8	Not detected		*C. tenuis*	37.5		
			*M. luteus*	9.4			*M. plumbeus*	75		
*Perenniporia centrali-africana*	Culture liquid	Isodrimenediol	Not detected		Not detected		*M. hiemalis*	67	Yes	[76]
							*R glutinis*	67		
*Porostereum spadiceum*	Mycelium	3,5-dichloro-4-methoxybenzaldehyde	*C michiganensis subsp*	100	*R. solanacearum*	100	*A. brassicicola*	100	Yes	[77]
							*C. orbiculare*	100		
*Psathyrella candolleana*	Culture liquid	Psathyrin A	*S. aureus*	14.3	*S. enterica*	77.9	Not investigated		No	[64].
		Psathyrin B	*S. aureus*	22.7	*S. enterica*	101.6	Not investigated			
*Skeletocutis* sp.	Mycelium, Culture liquid	Skeletocutin I	*B. subtilis*	18.75	Not detected		Not detected		Yes	[74]
			*S. aureus*	37.5						
*Skeletocutis* sp.	Mycelium, Culture liquid	Skeletocutin L	*B. subtilis*	18.75	Not detected		Not detected		Yes	[74]
			*M. luteus*	37.5						
			*S. aureus*	18.75						
		Tyromycin A	*B. subtilis*	9.375	Not detected		Not detected			
*Stereum hirsutum*	Culture liquid	Sterenin D	Not investigated		Not investigated		*B. cinerea*	20	No	[79]
*Tapinella atrotomentosa*	Fruiting bodies	Osmundalactone	Not detected		*A. baumannii*	10	Not investigated		No	[68]
					*E. coli*	10				
		5-hydroxy-hex-2-en-4-olide	Not detected		*A. baumannii*	6	Not investigated			
					*E. coli*	10				
					*M. catarrhalis*	50				
		Spiromentin C	Not detected		*A. baumannii*	20	Not investigated			
					*E. coli*	10				
					*M. catarrhalis*	50				

* SEQ—sequencing to determine the taxonomic position of a culture. **—The values are given in MIC_90_ (µg/mL). Not investigated—no testing had been conducted against this group of microorganisms. Not detected—no activity based on the results of article.

**Table 4:** Antimicrobial activity of compounds isolated from basidiomycetes (inhibition zone).

Species	Fungi Material	Compound	Inhibition Zone, mm					SEQ *	Reference
			Antibacterial Activity			Antifungal Activity			
			Gram-Positive Bacteria		Gram-Negative Bacteria				
*Coprinus rhizophorus*	Culture liquid	Sesquiterpene 2	*S. epidermidis*	13.8	Not detected	Not detected		No	[57]
		Echinolactone D	Not detected		Not detected	*S. cerevisiae*	38.6		
*Schizophyllum commune*	Culture liquid	Schizostatin	*S. aureus*	21.2	Not investigated	*A.solani*	20.2	Yes	[56]
						*Diaporte* sp.	19.5		

* SEQ—sequencing to determine the taxonomic position of a culture. Not investigated—no testing had been conducted against this group of microorganisms. Not detected—no activity based on the results of article.

## 4. Conclusions

Over the past 10 years, the number of scientific papers on the screening, isolation, and study of basidiomycete metabolites with antimicrobial properties has increased. Such metabolites have been isolated from the fruiting bodies, mycelium, or culture liquid of basidial fungi. The continuation of the study of antimicrobial metabolites isolated from fruiting bodies required the insulation of a pure culture of basidiomycete. Only this condition will ensure the reproducibility of the previously obtained results. An analysis of the articles used in this review showed that fruiting bodies were mainly used at the screening stage of the extracts. A molecular identification of the species had not been conducted in all works. Culture sequencing was conducted in 6 out of 16 articles on extracts and in 10 out of 18 articles on individual substances.

In all studies, testing of antibacterial and antifungal activity was carried out in in vitro experiments. The identified antimicrobial metabolites of basidiomycetes demonstrated a fairly wide range of activity, including Gram-positive and Gram-negative bacteria, mycelial and yeast-like fungi. The activity was detected against saprophytic microorganisms, human and animal pathogens, and phytopathogens. Collectible strains of bacteria and fungi and multi-resistant and clinical strains of pathogenic bacteria were the test objects. Antimicrobial activity could consist of inhibiting the growth of test objects, as well as inhibiting the formation of biofilms formed by bacteria and yeast-like fungi.

## Data Availability

Not applicable.
